# Supplementary figures and images for: Reduction of Cancer-Induced Thrombocytosis as a Biomarker of Improved Outcomes in Advanced Gastric Cancer
Source: J Clin Med. 2022 Feb 24;11(5):1213. doi: 10.3390/jcm11051213 (PMC8911022; doi:10.3390/jcm11051213)

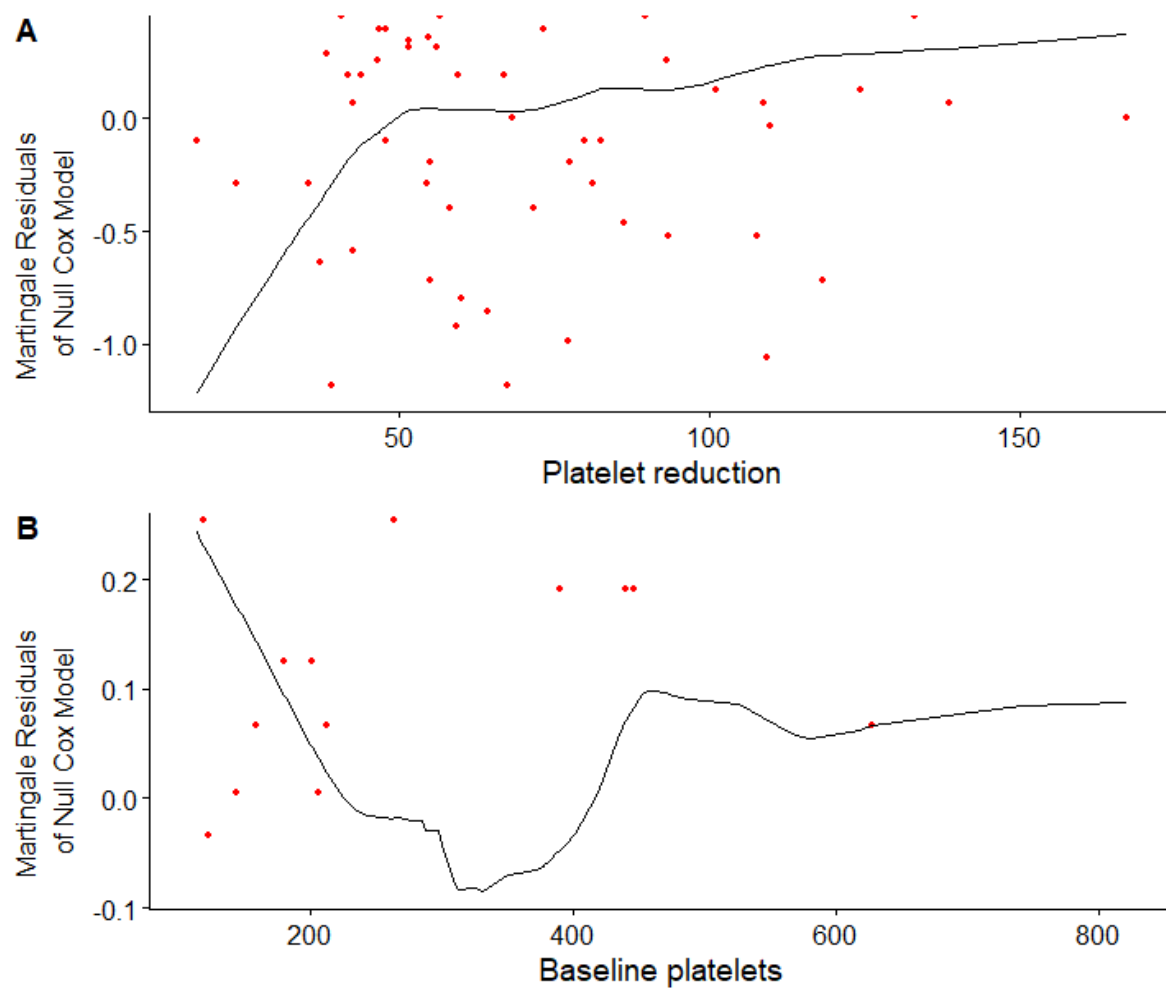

**Figure S1.** Relationship between PLT-red, PLT-count, and PFS.

Supplement: Supplementary file 1 [file jcm-11-01213-s001.zip › jcm-1609710-supplementary.pdf]
